# 4-[(2,4-Dihydroxy­benzyl­idene)ammonio]benzene­sulfonate trihydrate

**DOI:** 10.1107/S1600536810004526

**Published:** 2010-02-10

**Authors:** Chin Sing Yeap, Madhukar Hemamalini, Hoong-Kun Fun

**Affiliations:** a X-ray Crystallography Unit, School of Physics, Universiti Sains Malaysia, 11800 USM, Penang, Malaysia

## Abstract

The title Schiff base compound, C_13_H_11_NO_5_S·3H_2_O, formed from sulfanilic acid and 2,4-dihydroxy­benzaldehyde, crystallized out as a zwitterion with the N atom protonated. The asymmetric unit consists of one 4-[(2,4-dihydroxy­benzyl­idene)ammonio]benzene­sulfonate and three water mol­ecules. The zwitterion exists in an *E* configuration with respect to the central C=N double bond. The two benzene rings of the mol­ecule are oriented at a dihedral angle of 27.33 (8)°. An intra­molecular N–H⋯O hydrogen bond stabilizes the mol­ecular structure. In the crystal, the zwitterions are linked into chains along [101] by inter­molecular O—H⋯O and N—H⋯O hydrogen bonds. The three water mol­ecules link these chains into a three-dimensional framework by additional inter­molecular O—H⋯O hydrogen bonds. A π⋯π inter­action [3.5485 (9) Å] further stabilizes the crystal structure.

## Related literature

For Schiff bases and their applications, see: Singh *et al.* (1975 ▶); Elmali *et al.* (1999 ▶); Patel *et al.* (1999 ▶). For details of sulfanilic acid, see: Rae & Maslen (1962 ▶); Banu & Golzar Hossain (2006 ▶); Hempel *et al.* (1999 ▶). For hydrogen-bond motifs, see: Bernstein *et al.* (1995 ▶). For the stability of the temperature controller used for the data collection, see: Cosier & Glazer (1986 ▶).
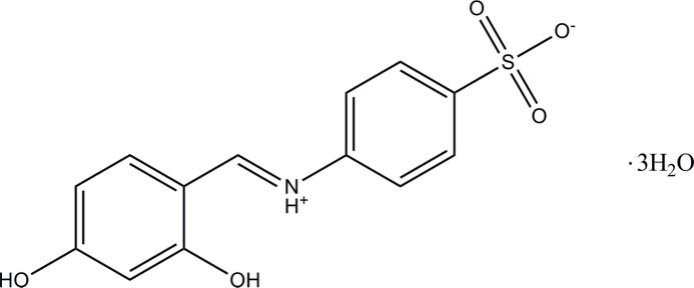


## Experimental

### 

#### Crystal data

C_13_H_11_NO_5_S·3H_2_O
*M* *_r_* = 347.34
Triclinic, 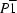

*a* = 7.7855 (1) Å
*b* = 9.0820 (1) Å
*c* = 11.8526 (2) Å
α = 70.022 (1)°
β = 79.271 (1)°
γ = 76.141 (1)°
*V* = 759.70 (2) Å^3^
*Z* = 2
Mo *K*α radiation
μ = 0.26 mm^−1^
*T* = 100 K
0.36 × 0.16 × 0.08 mm

#### Data collection

Bruker SMART APEXII CCD area-detector diffractometer
Absorption correction: multi-scan (*SADABS*; Bruker, 2009 ▶) *T*_min_ = 0.914, *T*_max_ = 0.980
19799 measured reflections
5420 independent reflections
4177 reflections with *I* > 2σ(*I*)
*R*_int_ = 0.027

#### Refinement

*R*[*F* ^2^ > 2σ(*F* ^2^)] = 0.053
*wR*(*F* ^2^) = 0.147
*S* = 1.05
5420 reflections
220 parameters
H atoms treated by a mixture of independent and constrained refinement
Δρ_max_ = 0.61 e Å^−3^
Δρ_min_ = −0.60 e Å^−3^

### 

Data collection: *APEX2* (Bruker, 2009 ▶); cell refinement: *SAINT* (Bruker, 2009 ▶); data reduction: *SAINT*; program(s) used to solve structure: *SHELXTL* (Sheldrick, 2008 ▶); program(s) used to refine structure: *SHELXTL*; molecular graphics: *SHELXTL*; software used to prepare material for publication: *SHELXTL* and *PLATON* (Spek, 2009 ▶).

## Supplementary Material

Crystal structure: contains datablocks global, I. DOI: 10.1107/S1600536810004526/sj2719sup1.cif

Structure factors: contains datablocks I. DOI: 10.1107/S1600536810004526/sj2719Isup2.hkl

Additional supplementary materials:  crystallographic information; 3D view; checkCIF report

## Figures and Tables

**Table 1:** Hydrogen-bond geometry (Å, °)

*D*—H⋯*A*	*D*—H	H⋯*A*	*D*⋯*A*	*D*—H⋯*A*
O1*W*—H1*W*1⋯O3^i^	0.89	2.17	3.020 (3)	161
O1*W*—H1*W*1⋯O4^i^	0.89	2.39	3.083 (3)	135
O1*W*—H2*W*1⋯O2*W*	0.84	2.02	2.817 (4)	158
O2*W*—H2*W*2⋯O2	0.95	1.89	2.812 (3)	161
O3*W*—H1*W*3⋯O5^ii^	0.96	1.83	2.756 (2)	161
O3*W*—H2*W*3⋯O1*W*	0.86	1.86	2.701 (3)	166
N1—H1*N*1⋯O1	0.86 (3)	2.07 (2)	2.6601 (18)	126 (2)
N1—H1*N*1⋯O5^iii^	0.86 (3)	2.19 (3)	2.948 (2)	148 (2)
O1—H1*O*1⋯O3*W*	0.90 (3)	1.64 (4)	2.543 (2)	173 (4)
O2—H1*O*2⋯O3^iv^	0.80 (3)	1.85 (3)	2.627 (2)	164 (2)

Symmetry codes: (i) 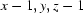; (ii) 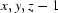; (iii) 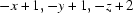; (iv) 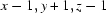.

**Fig. 1.**
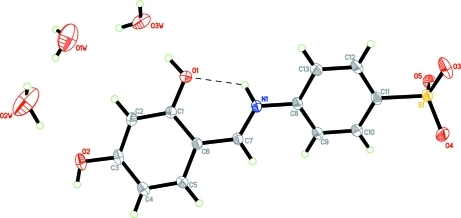
The molecular structure of (I) with atom labels and 50% probability ellipsoids for non-H atoms. The intramolecular hydrogen bond is shown as a dashed line.

**Fig. 2.**
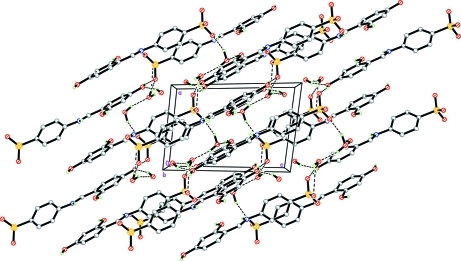
The crystal packing of (I), viewed down the b axis, showing the molecules linked into a 3-dimensional framework. Intermolecular hydrogen bonds are shown as dashed lines.

## supplementary crystallographic information

### Comment

Schiff bases derived from aromatic amines and aromatic aldehydes have a wide variety of applications in many fields, *e.g.*, biological, inorganic and analytical chemistry (Singh *et al.*, 1975; Elmali *et al.*, 1999; Patel *et al.*, 1999). Schiff base compounds have been of great interest for many years. *p*-aminobenzenesulfonic acid is known as sulfanilic acid, which contains NH_3_^+^ and SO_3_^-^ groups. Sulfanilic acid is a salt, but of a rather special kind, called a zwitterion. It is the product of the reaction between an acidic group and a basic group that are part of the same molecule. The hydrogen is attached to nitrogen rather than oxygen simply because the NH_2_ group is a stronger base than the SO_3_^-^ substituent. A zwitterionic structure was also observed in the crystal structure of sulfanilic acid monohydrate (Rae & Maslen, 1962; Banu & Golzar, 2006). The crystal structure of the Schiff base formed from sulfanilic acid and dimethylformamide has also been reported in the literature (Hempel *et al.*, 1999). The present work is part of a structural study of compounds of Schiff base systems and we report here the structure of the title compound, (I).

The 4-[(2,4-dihydroxybenzylidene)-amino]benzenesulfonic acid molecule crystallized out as a zwitterion, 4-[(2,4-dihydroxybenzylidene)-ammonio]benzenesulfonate. The asymmetric unit consists of one 4-[(2,4-dihydroxybenzylidene)-ammonio]benzenesulfonate and three water molecules (Fig. 1). The zwitterion exists in an *E* configuration with respect to the central C=N double bond. The two benzene rings [C1–C6 and C8–C13] are oriented at 27.33 (8)°. An intramolecular N1–H1N1···O1 hydrogen bond forms a six-membered ring, generating *S*(6) ring motif (Bernstein *et al.*, 1995). The zwitterions are linked into chains along [101] by intermolecular O2–H1O2···O3 and N1–H1N1···O5 hydrogen bonds. The three water molecules linked these chains into a three-dimensional framework by intermolecular O–H···O hydrogen bonds (Fig. 2, Table 2). An unusually short H2W1···H1W2 distance is also observed. A Cg1···Cg1 interaction of 3.5485 (9) Å; -x, 2-y, 1-z, further stabilizes the crystal structure [Cg1 is the centroid of the C1–C6 benzene ring].

### Experimental

2,4-Dihydroxybenzaldehyde (0.069 g) and sulfanilic acid (0.861 g) in ethanol/water (40 ml) were heated under reflux for 2 h with stirring. The colour of the solution gradually changed from colourless to lemon yellow. The solution was then cooled to room temperature. After few days, slow evaporation of the solvent yielded yellow crystals of compound (I).

### Refinement

The O and N bound H-atoms were located from difference Fourier map and refined freely. The H-atoms of the water molecules were located from a difference Fourier map and constrained to refine with the parent atom with U_iso_(H) = 1.5 U_eq_(O). The C-bound H-atoms were positioned geometrically with a riding model with C–H = 0.93 Å and U_iso_(H) = 1.2 U_eq_(C).

### Crystal data

**Table:**

C_13_H_11_NO_5_S·3H_2_O	*Z* = 2
*M**_r_* = 347.34	*F*(000) = 364
Triclinic, *P*1	*D*_x_ = 1.518 Mg m^−^^3^
Hall symbol: -P 1	Mo *K*α radiation, λ = 0.71073 Å
*a* = 7.7855 (1) Å	Cell parameters from 7701 reflections
*b* = 9.0820 (1) Å	θ = 2.6–32.1°
*c* = 11.8526 (2) Å	µ = 0.26 mm^−^^1^
α = 70.022 (1)°	*T* = 100 K
β = 79.271 (1)°	Block, yellow
γ = 76.141 (1)°	0.36 × 0.16 × 0.08 mm
*V* = 759.70 (2) Å^3^	

### Data collection

**Table:**

Bruker SMART APEXII CCD area-detector diffractometer	5420 independent reflections
Radiation source: fine-focus sealed tube	4177 reflections with *I* > 2σ(*I*)
graphite	*R*_int_ = 0.027
φ and ω scans	θ_max_ = 32.4°, θ_min_ = 1.8°
Absorption correction: multi-scan (*SADABS*; Bruker, 2009)	*h* = −11→11
*T*_min_ = 0.914, *T*_max_ = 0.980	*k* = −13→13
19799 measured reflections	*l* = −17→17

### Refinement

**Table:**

Refinement on *F*^2^	Primary atom site location: structure-invariant direct methods
Least-squares matrix: full	Secondary atom site location: difference Fourier map
*R*[*F*^2^ > 2σ(*F*^2^)] = 0.053	Hydrogen site location: inferred from neighbouring sites
*wR*(*F*^2^) = 0.147	H atoms treated by a mixture of independent and constrained refinement
*S* = 1.05	*w* = 1/[σ^2^(*F*_o_^2^) + (0.070*P*)^2^ + 0.4045*P*] where *P* = (*F*_o_^2^ + 2*F*_c_^2^)/3
5420 reflections	(Δ/σ)_max_ = 0.001
220 parameters	Δρ_max_ = 0.61 e Å^−^^3^
0 restraints	Δρ_min_ = −0.60 e Å^−^^3^

### Special details

**Table:**

Experimental. The crystal was placed in the cold stream of an Oxford Cryosystems Cobra open-flow nitrogen cryostat (Cosier & Glazer, 1986) operating at 100.0 (1) K.
Geometry. All esds (except the esd in the dihedral angle between two l.s. planes) are estimated using the full covariance matrix. The cell esds are taken into account individually in the estimation of esds in distances, angles and torsion angles; correlations between esds in cell parameters are only used when they are defined by crystal symmetry. An approximate (isotropic) treatment of cell esds is used for estimating esds involving l.s. planes.
Refinement. Refinement of *F*^2^ against ALL reflections. The weighted *R*-factor wR and goodness of fit *S* are based on *F*^2^, conventional *R*-factors *R* are based on *F*, with *F* set to zero for negative *F*^2^. The threshold expression of *F*^2^ > σ(*F*^2^) is used only for calculating *R*-factors(gt) etc. and is not relevant to the choice of reflections for refinement. *R*-factors based on *F*^2^ are statistically about twice as large as those based on *F*, and *R*- factors based on ALL data will be even larger.

### Fractional atomic coordinates and isotropic or equivalent isotropic displacement parameters (Å^2^)

**Table:**

	*x*	*y*	*z*	*U*_iso_*/*U*_eq_	
S1	0.71640 (6)	0.60676 (5)	1.18660 (4)	0.02504 (12)	
O1	0.33982 (17)	0.77778 (14)	0.52286 (11)	0.0226 (2)	
O2	0.00861 (17)	1.21132 (17)	0.24684 (11)	0.0256 (3)	
O3	0.89905 (19)	0.52191 (18)	1.16735 (13)	0.0371 (3)	
O4	0.7119 (3)	0.75067 (16)	1.21434 (16)	0.0530 (5)	
O5	0.60457 (18)	0.50241 (15)	1.27306 (10)	0.0267 (3)	
N1	0.41704 (17)	0.82152 (16)	0.71713 (11)	0.0170 (2)	
C1	0.2641 (2)	0.93133 (18)	0.48282 (13)	0.0170 (3)	
C2	0.1775 (2)	0.99664 (19)	0.37933 (13)	0.0190 (3)	
H2A	0.1728	0.9331	0.3333	0.023*	
C3	0.0980 (2)	1.15723 (19)	0.34488 (13)	0.0195 (3)	
C4	0.1103 (2)	1.25788 (19)	0.40959 (14)	0.0207 (3)	
H4A	0.0593	1.3658	0.3843	0.025*	
C5	0.1987 (2)	1.19458 (18)	0.51051 (14)	0.0188 (3)	
H5A	0.2090	1.2609	0.5528	0.023*	
C6	0.2748 (2)	1.02971 (17)	0.55143 (13)	0.0162 (3)	
C7	0.3521 (2)	0.97063 (18)	0.66134 (13)	0.0171 (3)	
H7A	0.3572	1.0454	0.6975	0.021*	
C8	0.4854 (2)	0.76888 (17)	0.83072 (13)	0.0169 (3)	
C9	0.4177 (2)	0.85045 (19)	0.91434 (14)	0.0212 (3)	
H9A	0.3246	0.9379	0.8979	0.025*	
C10	0.4905 (2)	0.79988 (19)	1.02271 (14)	0.0219 (3)	
H10A	0.4466	0.8537	1.0793	0.026*	
C11	0.6291 (2)	0.66864 (18)	1.04650 (14)	0.0197 (3)	
C12	0.6933 (2)	0.58417 (18)	0.96441 (14)	0.0203 (3)	
H12A	0.7847	0.4954	0.9817	0.024*	
C13	0.6196 (2)	0.63345 (18)	0.85627 (14)	0.0188 (3)	
H13A	0.6595	0.5765	0.8014	0.023*	
O1W	0.1128 (3)	0.7226 (3)	0.22066 (18)	0.0639 (6)	
H1W1	0.0299	0.6832	0.2044	0.096*	
H2W1	0.0754	0.8189	0.1836	0.096*	
O2W	0.0858 (3)	1.0446 (3)	0.0769 (2)	0.0815 (7)	
H1W2	0.1929	0.9994	0.0628	0.122*	
H2W2	0.0424	1.1170	0.1224	0.122*	
O3W	0.3001 (2)	0.59375 (18)	0.41308 (14)	0.0368 (3)	
H1W3	0.4180	0.5558	0.3796	0.055*	
H2W3	0.2254	0.6348	0.3604	0.055*	
H1N1	0.415 (3)	0.747 (3)	0.689 (2)	0.041 (7)*	
H1O1	0.327 (4)	0.719 (4)	0.478 (3)	0.057 (8)*	
H1O2	−0.027 (3)	1.305 (3)	0.236 (2)	0.036 (7)*	

### Atomic displacement parameters (Å^2^)

**Table:**

	*U*^11^	*U*^22^	*U*^33^	*U*^12^	*U*^13^	*U*^23^
S1	0.0369 (2)	0.01588 (18)	0.0241 (2)	−0.00608 (15)	−0.01801 (17)	−0.00021 (14)
O1	0.0310 (6)	0.0164 (5)	0.0212 (5)	−0.0008 (4)	−0.0084 (5)	−0.0063 (4)
O2	0.0257 (6)	0.0297 (7)	0.0188 (5)	−0.0058 (5)	−0.0101 (5)	−0.0001 (5)
O3	0.0291 (7)	0.0386 (8)	0.0338 (7)	−0.0045 (6)	−0.0170 (6)	0.0066 (6)
O4	0.0943 (14)	0.0183 (6)	0.0590 (10)	−0.0066 (7)	−0.0542 (10)	−0.0072 (6)
O5	0.0367 (7)	0.0239 (6)	0.0173 (5)	−0.0029 (5)	−0.0064 (5)	−0.0037 (4)
N1	0.0192 (6)	0.0167 (6)	0.0146 (5)	−0.0028 (5)	−0.0048 (4)	−0.0031 (5)
C1	0.0172 (6)	0.0175 (6)	0.0165 (6)	−0.0047 (5)	−0.0019 (5)	−0.0047 (5)
C2	0.0198 (7)	0.0228 (7)	0.0158 (6)	−0.0070 (5)	−0.0033 (5)	−0.0051 (5)
C3	0.0176 (7)	0.0241 (7)	0.0145 (6)	−0.0066 (5)	−0.0037 (5)	−0.0004 (5)
C4	0.0208 (7)	0.0186 (7)	0.0195 (7)	−0.0035 (5)	−0.0043 (6)	−0.0008 (5)
C5	0.0219 (7)	0.0161 (6)	0.0175 (6)	−0.0034 (5)	−0.0042 (5)	−0.0033 (5)
C6	0.0177 (6)	0.0159 (6)	0.0145 (6)	−0.0032 (5)	−0.0035 (5)	−0.0031 (5)
C7	0.0193 (7)	0.0165 (6)	0.0152 (6)	−0.0035 (5)	−0.0047 (5)	−0.0031 (5)
C8	0.0174 (6)	0.0165 (6)	0.0150 (6)	−0.0039 (5)	−0.0044 (5)	−0.0012 (5)
C9	0.0247 (7)	0.0184 (7)	0.0182 (7)	0.0005 (6)	−0.0065 (6)	−0.0039 (5)
C10	0.0288 (8)	0.0176 (7)	0.0183 (7)	−0.0018 (6)	−0.0067 (6)	−0.0038 (5)
C11	0.0234 (7)	0.0158 (6)	0.0194 (7)	−0.0068 (5)	−0.0082 (6)	0.0004 (5)
C12	0.0192 (7)	0.0170 (7)	0.0211 (7)	−0.0025 (5)	−0.0063 (5)	−0.0002 (5)
C13	0.0195 (7)	0.0176 (6)	0.0178 (6)	−0.0031 (5)	−0.0025 (5)	−0.0038 (5)
O1W	0.0496 (11)	0.1022 (17)	0.0610 (12)	−0.0363 (11)	−0.0095 (9)	−0.0358 (12)
O2W	0.0746 (15)	0.120 (2)	0.0831 (16)	−0.0358 (15)	0.0051 (12)	−0.0697 (16)
O3W	0.0402 (8)	0.0364 (7)	0.0420 (8)	−0.0097 (6)	0.0007 (6)	−0.0238 (6)

### Geometric parameters (Å, °)

**Table:**

S1—O4	1.4441 (14)	C5—H5A	0.9300
S1—O5	1.4512 (14)	C6—C7	1.417 (2)
S1—O3	1.4636 (15)	C7—H7A	0.9300
S1—C11	1.7706 (16)	C8—C9	1.389 (2)
O1—C1	1.3325 (18)	C8—C13	1.394 (2)
O1—H1O1	0.90 (3)	C9—C10	1.388 (2)
O2—C3	1.3500 (18)	C9—H9A	0.9300
O2—H1O2	0.80 (3)	C10—C11	1.390 (2)
N1—C7	1.3083 (19)	C10—H10A	0.9300
N1—C8	1.4247 (19)	C11—C12	1.389 (2)
N1—H1N1	0.85 (3)	C12—C13	1.390 (2)
C1—C2	1.389 (2)	C12—H12A	0.9300
C1—C6	1.422 (2)	C13—H13A	0.9300
C2—C3	1.390 (2)	O1W—H1W1	0.8863
C2—H2A	0.9300	O1W—H2W1	0.8403
C3—C4	1.408 (2)	O2W—H1W2	0.8500
C4—C5	1.372 (2)	O2W—H2W2	0.9519
C4—H4A	0.9300	O3W—H1W3	0.9617
C5—C6	1.419 (2)	O3W—H2W3	0.8594
			
O4—S1—O5	113.78 (10)	C7—C6—C1	123.35 (13)
O4—S1—O3	111.65 (11)	C5—C6—C1	118.67 (13)
O5—S1—O3	111.87 (8)	N1—C7—C6	126.60 (14)
O4—S1—C11	106.23 (8)	N1—C7—H7A	116.7
O5—S1—C11	106.54 (8)	C6—C7—H7A	116.7
O3—S1—C11	106.16 (8)	C9—C8—C13	121.03 (14)
C1—O1—H1O1	114.3 (19)	C9—C8—N1	120.33 (13)
C3—O2—H1O2	106.3 (18)	C13—C8—N1	118.64 (13)
C7—N1—C8	123.83 (13)	C10—C9—C8	119.21 (14)
C7—N1—H1N1	121.2 (17)	C10—C9—H9A	120.4
C8—N1—H1N1	114.9 (17)	C8—C9—H9A	120.4
O1—C1—C2	123.11 (14)	C9—C10—C11	119.89 (15)
O1—C1—C6	116.94 (13)	C9—C10—H10A	120.1
C2—C1—C6	119.94 (14)	C11—C10—H10A	120.1
C1—C2—C3	119.77 (14)	C12—C11—C10	120.89 (14)
C1—C2—H2A	120.1	C12—C11—S1	120.66 (12)
C3—C2—H2A	120.1	C10—C11—S1	118.41 (12)
O2—C3—C2	116.77 (15)	C11—C12—C13	119.41 (14)
O2—C3—C4	121.90 (15)	C11—C12—H12A	120.3
C2—C3—C4	121.33 (14)	C13—C12—H12A	120.3
C5—C4—C3	119.07 (14)	C12—C13—C8	119.48 (14)
C5—C4—H4A	120.5	C12—C13—H13A	120.3
C3—C4—H4A	120.5	C8—C13—H13A	120.3
C4—C5—C6	121.14 (14)	H1W1—O1W—H2W1	97.0
C4—C5—H5A	119.4	H1W2—O2W—H2W2	127.8
C6—C5—H5A	119.4	H1W3—O3W—H2W3	113.2
C7—C6—C5	117.94 (13)		
			
O1—C1—C2—C3	178.43 (14)	C7—N1—C8—C13	−150.63 (15)
C6—C1—C2—C3	−1.5 (2)	C13—C8—C9—C10	2.8 (2)
C1—C2—C3—O2	−176.86 (14)	N1—C8—C9—C10	−177.92 (15)
C1—C2—C3—C4	3.1 (2)	C8—C9—C10—C11	−0.2 (2)
O2—C3—C4—C5	178.14 (14)	C9—C10—C11—C12	−1.7 (3)
C2—C3—C4—C5	−1.8 (2)	C9—C10—C11—S1	−179.29 (13)
C3—C4—C5—C6	−1.1 (2)	O4—S1—C11—C12	146.08 (15)
C4—C5—C6—C7	−175.27 (15)	O5—S1—C11—C12	−92.28 (15)
C4—C5—C6—C1	2.5 (2)	O3—S1—C11—C12	27.10 (16)
O1—C1—C6—C7	−3.5 (2)	O4—S1—C11—C10	−36.33 (17)
C2—C1—C6—C7	176.45 (14)	O5—S1—C11—C10	85.31 (14)
O1—C1—C6—C5	178.82 (14)	O3—S1—C11—C10	−155.30 (14)
C2—C1—C6—C5	−1.2 (2)	C10—C11—C12—C13	1.1 (2)
C8—N1—C7—C6	−176.86 (14)	S1—C11—C12—C13	178.61 (12)
C5—C6—C7—N1	174.98 (15)	C11—C12—C13—C8	1.5 (2)
C1—C6—C7—N1	−2.7 (3)	C9—C8—C13—C12	−3.4 (2)
C7—N1—C8—C9	30.1 (2)	N1—C8—C13—C12	177.29 (14)

### Hydrogen-bond geometry (Å, °)

**Table:**

*D*—H···*A*	*D*—H	H···*A*	*D*···*A*	*D*—H···*A*
O1W—H1W1···O3^i^	0.89	2.17	3.020 (3)	161
O1W—H1W1···O4^i^	0.89	2.39	3.083 (3)	135
O1W—H2W1···O2W	0.84	2.02	2.817 (4)	158
O2W—H2W2···O2	0.95	1.89	2.812 (3)	161
O3W—H1W3···O5^ii^	0.96	1.83	2.756 (2)	161
O3W—H2W3···O1W	0.86	1.86	2.701 (3)	166
N1—H1N1···O1	0.86 (3)	2.07 (2)	2.6601 (18)	126 (2)
N1—H1N1···O5^iii^	0.86 (3)	2.19 (3)	2.948 (2)	148 (2)
O1—H1O1···O3W	0.90 (3)	1.64 (4)	2.543 (2)	173 (4)
O2—H1O2···O3^iv^	0.80 (3)	1.85 (3)	2.627 (2)	164 (2)

Symmetry codes: (i) *x*−1, *y*, *z*−1; (ii) *x*, *y*, *z*−1; (iii) −*x*+1, −*y*+1, −*z*+2; (iv) *x*−1, *y*+1, *z*−1.
